# Supplementary material for: Modeling glioblastoma heterogeneity as a dynamic network of cell states
Source: Mol Syst Biol. 2021 Sep 16;17(9):e10105. doi: 10.15252/msb.202010105 (PMC8444284; doi:10.15252/msb.202010105)
Supplement: Supplementary file 6 — Source Data for Figure 5 [file MSB-17-e10105-s004.zip › Figure5A_sourcedata/GSEA_3071/nosigdb_stateIV.GseaPreranked.1622795164626/RICHARDS_NOWAKOWSKI_SCIENCE_2017_NEN_EARLY2_UPREG.html]

Details for gene set RICHARDS\_NOWAKOWSKI\_SCIENCE\_2017\_NEN\_EARLY2\_UPREG[GSEA]

|  || Dataset | state53071 |
| Phenotype | NoPhenotypeAvailable |
| Upregulated in class | na\_pos |
| GeneSet | RICHARDS\_NOWAKOWSKI\_SCIENCE\_2017\_NEN\_EARLY2\_UPREG |
| Enrichment Score (ES) | 0.44101533 |
| Normalized Enrichment Score (NES) | 2.810452 |
| Nominal p-value | 0.0 |
| FDR q-value | 0.0 |
| FWER p-Value | 0.0 |
Table: GSEA Results Summary

  

Fig 1: Enrichment plot: RICHARDS\_NOWAKOWSKI\_SCIENCE\_2017\_NEN\_EARLY2\_UPREG      
 Profile of the Running ES Score & Positions of GeneSet Members on the Rank Ordered List

  

| PROBE | GENE SYMBOL | GENE\_TITLE | RANK IN GENE LIST | RANK METRIC SCORE | RUNNING ES | CORE ENRICHMENT || 1 | BEX1 |  |  | 18 | 2.252 | 0.0077 | Yes |
| 2 | PLXNA4 |  |  | 20 | 2.236 | 0.0188 | Yes |
| 3 | MEIS2 |  |  | 32 | 2.004 | 0.0266 | Yes |
| 4 | SORL1 |  |  | 45 | 1.872 | 0.0336 | Yes |
| 5 | SLC1A2 |  |  | 48 | 1.860 | 0.0426 | Yes |
| 6 | POU3F3 |  |  | 51 | 1.857 | 0.0515 | Yes |
| 7 | MAP2 |  |  | 55 | 1.811 | 0.0600 | Yes |
| 8 | SOX11 |  |  | 56 | 1.811 | 0.0691 | Yes |
| 9 | TUBB2B |  |  | 69 | 1.731 | 0.0754 | Yes |
| 10 | POU3F2 |  |  | 70 | 1.728 | 0.0841 | Yes |
| 11 | TAGLN3 |  |  | 71 | 1.720 | 0.0927 | Yes |
| 12 | PTPRO |  |  | 80 | 1.628 | 0.0993 | Yes |
| 13 | EML6 |  |  | 84 | 1.619 | 0.1069 | Yes |
| 14 | PDP1 |  |  | 89 | 1.579 | 0.1140 | Yes |
| 15 | PGAP1 |  |  | 94 | 1.556 | 0.1210 | Yes |
| 16 | GPM6A |  |  | 98 | 1.538 | 0.1281 | Yes |
| 17 | FRMD4B |  |  | 106 | 1.519 | 0.1344 | Yes |
| 18 | AFF3 |  |  | 109 | 1.510 | 0.1416 | Yes |
| 19 | ROBO2 |  |  | 114 | 1.499 | 0.1483 | Yes |
| 20 | NFIB |  |  | 117 | 1.491 | 0.1554 | Yes |
| 21 | TTC28 |  |  | 119 | 1.490 | 0.1627 | Yes |
| 22 | APP |  |  | 136 | 1.426 | 0.1666 | Yes |
| 23 | CXADR |  |  | 139 | 1.402 | 0.1733 | Yes |
| 24 | TIAM2 |  |  | 141 | 1.393 | 0.1801 | Yes |
| 25 | GRIA2 |  |  | 160 | 1.347 | 0.1833 | Yes |
| 26 | NFIX |  |  | 162 | 1.345 | 0.1898 | Yes |
| 27 | TUBA1A |  |  | 165 | 1.337 | 0.1961 | Yes |
| 28 | TUBB3 |  |  | 167 | 1.330 | 0.2026 | Yes |
| 29 | PLCB1 |  |  | 168 | 1.323 | 0.2093 | Yes |
| 30 | TNIK |  |  | 172 | 1.312 | 0.2153 | Yes |
| 31 | IGSF3 |  |  | 173 | 1.309 | 0.2218 | Yes |
| 32 | CSRP2 |  |  | 175 | 1.305 | 0.2282 | Yes |
| 33 | GRIA3 |  |  | 191 | 1.277 | 0.2316 | Yes |
| 34 | MCF2L |  |  | 194 | 1.272 | 0.2376 | Yes |
| 35 | MEIS3 |  |  | 205 | 1.253 | 0.2419 | Yes |
| 36 | MYEF2 |  |  | 225 | 1.196 | 0.2441 | Yes |
| 37 | KIF5C |  |  | 240 | 1.170 | 0.2472 | Yes |
| 38 | HES6 |  |  | 250 | 1.150 | 0.2512 | Yes |
| 39 | HUNK |  |  | 251 | 1.146 | 0.2569 | Yes |
| 40 | IRGQ |  |  | 261 | 1.122 | 0.2608 | Yes |
| 41 | FOXO3 |  |  | 291 | 1.054 | 0.2603 | Yes |
| 42 | MEX3A |  |  | 296 | 1.038 | 0.2647 | Yes |
| 43 | BICD1 |  |  | 314 | 1.020 | 0.2664 | Yes |
| 44 | ARIH1 |  |  | 324 | 1.000 | 0.2697 | Yes |
| 45 | GTF2I |  |  | 329 | 0.990 | 0.2738 | Yes |
| 46 | FOXN3 |  |  | 348 | 0.977 | 0.2752 | Yes |
| 47 | KIF5A |  |  | 358 | 0.961 | 0.2782 | Yes |
| 48 | FOXG1 |  |  | 363 | 0.950 | 0.2822 | Yes |
| 49 | CTTNBP2 |  |  | 369 | 0.942 | 0.2859 | Yes |
| 50 | ST8SIA1 |  |  | 379 | 0.926 | 0.2888 | Yes |
| 51 | TOP2B |  |  | 382 | 0.924 | 0.2930 | Yes |
| 52 | MAP3K13 |  |  | 388 | 0.917 | 0.2966 | Yes |
| 53 | ATCAY |  |  | 397 | 0.910 | 0.2996 | Yes |
| 54 | DPYSL5 |  |  | 411 | 0.895 | 0.3015 | Yes |
| 55 | GSE1 |  |  | 418 | 0.885 | 0.3047 | Yes |
| 56 | KLHL24 |  |  | 419 | 0.885 | 0.3092 | Yes |
| 57 | CELSR2 |  |  | 428 | 0.875 | 0.3120 | Yes |
| 58 | PREX1 |  |  | 429 | 0.873 | 0.3164 | Yes |
| 59 | PDE9A |  |  | 433 | 0.868 | 0.3201 | Yes |
| 60 | KDM5B |  |  | 434 | 0.868 | 0.3245 | Yes |
| 61 | CRMP1 |  |  | 438 | 0.866 | 0.3282 | Yes |
| 62 | CYFIP2 |  |  | 450 | 0.856 | 0.3303 | Yes |
| 63 | CHRDL1 |  |  | 452 | 0.854 | 0.3344 | Yes |
| 64 | AGO1 |  |  | 453 | 0.853 | 0.3387 | Yes |
| 65 | MLXIP |  |  | 454 | 0.851 | 0.3430 | Yes |
| 66 | ANTXR1 |  |  | 462 | 0.847 | 0.3459 | Yes |
| 67 | RTN1 |  |  | 471 | 0.836 | 0.3485 | Yes |
| 68 | DCC |  |  | 472 | 0.835 | 0.3527 | Yes |
| 69 | ZNF608 |  |  | 478 | 0.832 | 0.3558 | Yes |
| 70 | LDLRAD4 |  |  | 496 | 0.819 | 0.3566 | Yes |
| 71 | ZNF704 |  |  | 498 | 0.818 | 0.3605 | Yes |
| 72 | ZKSCAN1 |  |  | 524 | 0.794 | 0.3595 | Yes |
| 73 | B4GALT5 |  |  | 541 | 0.774 | 0.3602 | Yes |
| 74 | CD24 |  |  | 545 | 0.770 | 0.3634 | Yes |
| 75 | ADD2 |  |  | 572 | 0.747 | 0.3620 | Yes |
| 76 | FNBP1L |  |  | 576 | 0.741 | 0.3651 | Yes |
| 77 | ELAVL3 |  |  | 582 | 0.739 | 0.3678 | Yes |
| 78 | ZBTB18 |  |  | 587 | 0.736 | 0.3707 | Yes |
| 79 | TSC22D1 |  |  | 588 | 0.735 | 0.3744 | Yes |
| 80 | ENC1 |  |  | 590 | 0.734 | 0.3779 | Yes |
| 81 | ENAH |  |  | 601 | 0.726 | 0.3796 | Yes |
| 82 | SLC29A4 |  |  | 603 | 0.726 | 0.3830 | Yes |
| 83 | CDH4 |  |  | 610 | 0.721 | 0.3854 | Yes |
| 84 | CECR2 |  |  | 612 | 0.718 | 0.3889 | Yes |
| 85 | ZNF506 |  |  | 615 | 0.716 | 0.3921 | Yes |
| 86 | BRD3 |  |  | 626 | 0.710 | 0.3936 | Yes |
| 87 | ARHGAP21 |  |  | 636 | 0.706 | 0.3954 | Yes |
| 88 | DDAH2 |  |  | 655 | 0.686 | 0.3952 | Yes |
| 89 | CALCOCO1 |  |  | 683 | 0.671 | 0.3932 | Yes |
| 90 | TNRC6B |  |  | 698 | 0.662 | 0.3938 | Yes |
| 91 | NREP |  |  | 701 | 0.660 | 0.3967 | Yes |
| 92 | TRAK1 |  |  | 717 | 0.650 | 0.3969 | Yes |
| 93 | IP6K2 |  |  | 722 | 0.647 | 0.3994 | Yes |
| 94 | NHSL1 |  |  | 734 | 0.641 | 0.4004 | Yes |
| 95 | PGM2L1 |  |  | 735 | 0.640 | 0.4036 | Yes |
| 96 | C14orf132 |  |  | 758 | 0.624 | 0.4024 | Yes |
| 97 | SLC22A17 |  |  | 762 | 0.623 | 0.4049 | Yes |
| 98 | PPP1R9A |  |  | 768 | 0.621 | 0.4070 | Yes |
| 99 | KIDINS220 |  |  | 777 | 0.616 | 0.4085 | Yes |
| 100 | ST3GAL6 |  |  | 785 | 0.610 | 0.4102 | Yes |
| 101 | SRGAP3 |  |  | 789 | 0.609 | 0.4127 | Yes |
| 102 | SLC44A5 |  |  | 810 | 0.599 | 0.4117 | Yes |
| 103 | PRKX |  |  | 815 | 0.597 | 0.4139 | Yes |
| 104 | SEMA6D |  |  | 830 | 0.589 | 0.4141 | Yes |
| 105 | CREBBP |  |  | 848 | 0.581 | 0.4136 | Yes |
| 106 | RSBN1L |  |  | 870 | 0.573 | 0.4123 | Yes |
| 107 | GTF2IRD2 |  |  | 878 | 0.569 | 0.4137 | Yes |
| 108 | PAG1 |  |  | 882 | 0.568 | 0.4160 | Yes |
| 109 | ZNF195 |  |  | 883 | 0.568 | 0.4189 | Yes |
| 110 | KIF3C |  |  | 885 | 0.567 | 0.4215 | Yes |
| 111 | STX6 |  |  | 910 | 0.557 | 0.4195 | Yes |
| 112 | CEP170 |  |  | 935 | 0.548 | 0.4175 | Yes |
| 113 | SYP |  |  | 955 | 0.541 | 0.4164 | Yes |
| 114 | SCG5 |  |  | 961 | 0.539 | 0.4181 | Yes |
| 115 | EIF1B |  |  | 978 | 0.533 | 0.4176 | Yes |
| 116 | NUDT4 |  |  | 991 | 0.525 | 0.4178 | Yes |
| 117 | EPHA7 |  |  | 997 | 0.523 | 0.4195 | Yes |
| 118 | RABGAP1 |  |  | 998 | 0.523 | 0.4221 | Yes |
| 119 | CLASP2 |  |  | 1017 | 0.515 | 0.4211 | Yes |
| 120 | ZNF286A |  |  | 1020 | 0.514 | 0.4233 | Yes |
| 121 | ZBTB10 |  |  | 1027 | 0.513 | 0.4247 | Yes |
| 122 | GATAD2B |  |  | 1030 | 0.512 | 0.4268 | Yes |
| 123 | CCDC88C |  |  | 1032 | 0.511 | 0.4292 | Yes |
| 124 | APLP1 |  |  | 1035 | 0.510 | 0.4314 | Yes |
| 125 | LUC7L |  |  | 1037 | 0.509 | 0.4337 | Yes |
| 126 | EVL |  |  | 1047 | 0.506 | 0.4345 | Yes |
| 127 | TSPAN14 |  |  | 1056 | 0.504 | 0.4354 | Yes |
| 128 | SORBS1 |  |  | 1099 | 0.492 | 0.4295 | Yes |
| 129 | JMJD1C |  |  | 1108 | 0.488 | 0.4304 | Yes |
| 130 | ZNF462 |  |  | 1111 | 0.488 | 0.4324 | Yes |
| 131 | BSDC1 |  |  | 1139 | 0.483 | 0.4295 | Yes |
| 132 | IGF1R |  |  | 1140 | 0.482 | 0.4319 | Yes |
| 133 | PRRC2B |  |  | 1152 | 0.479 | 0.4321 | Yes |
| 134 | ZNF292 |  |  | 1153 | 0.479 | 0.4345 | Yes |
| 135 | VASH1 |  |  | 1165 | 0.476 | 0.4347 | Yes |
| 136 | NAV1 |  |  | 1170 | 0.474 | 0.4363 | Yes |
| 137 | NEUROD1 |  |  | 1180 | 0.471 | 0.4369 | Yes |
| 138 | AUTS2 |  |  | 1211 | 0.462 | 0.4332 | Yes |
| 139 | LONRF1 |  |  | 1213 | 0.462 | 0.4353 | Yes |
| 140 | GNG4 |  |  | 1230 | 0.457 | 0.4344 | Yes |
| 141 | DOCK4 |  |  | 1247 | 0.452 | 0.4335 | Yes |
| 142 | PTPRS |  |  | 1253 | 0.451 | 0.4348 | Yes |
| 143 | SLC4A7 |  |  | 1292 | 0.441 | 0.4294 | Yes |
| 144 | ATP6V0A1 |  |  | 1320 | 0.436 | 0.4262 | Yes |
| 145 | SECISBP2 |  |  | 1321 | 0.435 | 0.4284 | Yes |
| 146 | SSBP3 |  |  | 1342 | 0.430 | 0.4266 | Yes |
| 147 | ARFGEF1 |  |  | 1353 | 0.428 | 0.4267 | Yes |
| 148 | HIVEP3 |  |  | 1354 | 0.427 | 0.4289 | Yes |
| 149 | PHF20L1 |  |  | 1360 | 0.426 | 0.4300 | Yes |
| 150 | EPHA3 |  |  | 1362 | 0.425 | 0.4319 | Yes |
| 151 | ST6GAL1 |  |  | 1380 | 0.421 | 0.4307 | Yes |
| 152 | FOXN2 |  |  | 1381 | 0.421 | 0.4328 | Yes |
| 153 | STT3B |  |  | 1394 | 0.417 | 0.4325 | Yes |
| 154 | CAP2 |  |  | 1410 | 0.414 | 0.4316 | Yes |
| 155 | CSNK1E |  |  | 1412 | 0.413 | 0.4335 | Yes |
| 156 | CEP85L |  |  | 1413 | 0.413 | 0.4355 | Yes |
| 157 | AKT3 |  |  | 1417 | 0.412 | 0.4370 | Yes |
| 158 | SOX5 |  |  | 1422 | 0.412 | 0.4383 | Yes |
| 159 | B3GALT1 |  |  | 1430 | 0.409 | 0.4389 | Yes |
| 160 | KLF3 |  |  | 1437 | 0.406 | 0.4398 | Yes |
| 161 | STRBP |  |  | 1451 | 0.402 | 0.4392 | Yes |
| 162 | RB1CC1 |  |  | 1453 | 0.401 | 0.4410 | Yes |
| 163 | FYN |  |  | 1483 | 0.394 | 0.4372 | No |
| 164 | PPFIA2 |  |  | 1516 | 0.385 | 0.4328 | No |
| 165 | CAMK4 |  |  | 1518 | 0.385 | 0.4345 | No |
| 166 | RSF1 |  |  | 1521 | 0.384 | 0.4360 | No |
| 167 | ZNF562 |  |  | 1533 | 0.382 | 0.4358 | No |
| 168 | ARHGEF7 |  |  | 1536 | 0.381 | 0.4373 | No |
| 169 | CEP97 |  |  | 1556 | 0.376 | 0.4354 | No |
| 170 | ZNF3 |  |  | 1567 | 0.374 | 0.4353 | No |
| 171 | FAM222B |  |  | 1570 | 0.373 | 0.4367 | No |
| 172 | NFASC |  |  | 1618 | 0.364 | 0.4292 | No |
| 173 | AKAP6 |  |  | 1627 | 0.363 | 0.4294 | No |
| 174 | SREBF2 |  |  | 1681 | 0.353 | 0.4206 | No |
| 175 | PTPN4 |  |  | 1682 | 0.353 | 0.4224 | No |
| 176 | NARF |  |  | 1692 | 0.351 | 0.4224 | No |
| 177 | PRSS12 |  |  | 1704 | 0.349 | 0.4219 | No |
| 178 | ACVR2B |  |  | 1725 | 0.345 | 0.4197 | No |
| 179 | ZC2HC1A |  |  | 1730 | 0.344 | 0.4206 | No |
| 180 | FBXL20 |  |  | 1732 | 0.343 | 0.4221 | No |
| 181 | GPR137C |  |  | 1742 | 0.341 | 0.4220 | No |
| 182 | TM2D3 |  |  | 1747 | 0.340 | 0.4229 | No |
| 183 | HECTD4 |  |  | 1764 | 0.337 | 0.4214 | No |
| 184 | MKLN1 |  |  | 1769 | 0.336 | 0.4223 | No |
| 185 | LENG8 |  |  | 1779 | 0.335 | 0.4222 | No |
| 186 | ZNF160 |  |  | 1780 | 0.334 | 0.4239 | No |
| 187 | SENP5 |  |  | 1815 | 0.328 | 0.4188 | No |
| 188 | NDUFA13 |  |  | 1828 | 0.327 | 0.4180 | No |
| 189 | ATXN7L3B |  |  | 1844 | 0.325 | 0.4167 | No |
| 190 | FAM117B |  |  | 1853 | 0.323 | 0.4167 | No |
| 191 | ANKRD44 |  |  | 1875 | 0.317 | 0.4141 | No |
| 192 | YEATS2 |  |  | 1885 | 0.316 | 0.4139 | No |
| 193 | OCIAD2 |  |  | 1906 | 0.313 | 0.4115 | No |
| 194 | ZNF124 |  |  | 1926 | 0.309 | 0.4092 | No |
| 195 | ELAVL2 |  |  | 1938 | 0.307 | 0.4086 | No |
| 196 | ELMO1 |  |  | 1966 | 0.303 | 0.4047 | No |
| 197 | EPB41L5 |  |  | 1969 | 0.303 | 0.4058 | No |
| 198 | SPAST |  |  | 1986 | 0.300 | 0.4042 | No |
| 199 | BRAF |  |  | 1992 | 0.298 | 0.4047 | No |
| 200 | ZNF84 |  |  | 1994 | 0.298 | 0.4060 | No |
| 201 | LINC01102 |  |  | 2020 | 0.293 | 0.4024 | No |
| 202 | GLCE |  |  | 2028 | 0.292 | 0.4025 | No |
| 203 | NAB1 |  |  | 2031 | 0.292 | 0.4036 | No |
| 204 | EPHA4 |  |  | 2032 | 0.292 | 0.4050 | No |
| 205 | SNCAIP |  |  | 2043 | 0.290 | 0.4045 | No |
| 206 | SEZ6L |  |  | 2065 | 0.287 | 0.4018 | No |
| 207 | PUM2 |  |  | 2099 | 0.284 | 0.3966 | No |
| 208 | PDE4D |  |  | 2100 | 0.283 | 0.3980 | No |
| 209 | CDK5R1 |  |  | 2126 | 0.280 | 0.3945 | No |
| 210 | MORC3 |  |  | 2140 | 0.279 | 0.3933 | No |
| 211 | SOX12 |  |  | 2141 | 0.278 | 0.3947 | No |
| 212 | ARPP21 |  |  | 2257 | 0.261 | 0.3730 | No |
| 213 | ZNF8 |  |  | 2300 | 0.255 | 0.3659 | No |
| 214 | SPIN1 |  |  | 2358 | -0.254 | 0.3558 | No |
| 215 | ZMIZ1 |  |  | 2414 | -0.259 | 0.3462 | No |
| 216 | SEC63 |  |  | 2420 | -0.260 | 0.3465 | No |
| 217 | TRIT1 |  |  | 2440 | -0.262 | 0.3440 | No |
| 218 | L3MBTL3 |  |  | 2441 | -0.262 | 0.3453 | No |
| 219 | KIAA0232 |  |  | 2529 | -0.272 | 0.3293 | No |
| 220 | PTPRD |  |  | 2553 | -0.275 | 0.3261 | No |
| 221 | HIVEP2 |  |  | 2558 | -0.276 | 0.3267 | No |
| 222 | PPP2R1B |  |  | 2639 | -0.288 | 0.3122 | No |
| 223 | FNBP1 |  |  | 2706 | -0.295 | 0.3005 | No |
| 224 | TTC39C |  |  | 2723 | -0.296 | 0.2988 | No |
| 225 | LARP1 |  |  | 2816 | -0.307 | 0.2820 | No |
| 226 | LAMB1 |  |  | 3029 | -0.336 | 0.2414 | No |
| 227 | ASXL3 |  |  | 3111 | -0.348 | 0.2270 | No |
| 228 | SPIRE1 |  |  | 3141 | -0.352 | 0.2230 | No |
| 229 | ANK3 |  |  | 3216 | -0.363 | 0.2101 | No |
| 230 | SVIL |  |  | 3218 | -0.363 | 0.2117 | No |
| 231 | PDHA1 |  |  | 3244 | -0.367 | 0.2086 | No |
| 232 | PRDM8 |  |  | 3249 | -0.368 | 0.2096 | No |
| 233 | RNF144A |  |  | 3276 | -0.371 | 0.2063 | No |
| 234 | RPL36A |  |  | 3280 | -0.372 | 0.2076 | No |
| 235 | EML1 |  |  | 3297 | -0.374 | 0.2063 | No |
| 236 | ARPC5 |  |  | 3388 | -0.388 | 0.1903 | No |
| 237 | EPHB6 |  |  | 3391 | -0.389 | 0.1918 | No |
| 238 | KPNA3 |  |  | 3423 | -0.394 | 0.1876 | No |
| 239 | PSMG2 |  |  | 3459 | -0.401 | 0.1826 | No |
| 240 | IRS1 |  |  | 3584 | -0.424 | 0.1600 | No |
| 241 | ZPR1 |  |  | 3604 | -0.429 | 0.1584 | No |
| 242 | CDC42EP3 |  |  | 3608 | -0.430 | 0.1600 | No |
| 243 | ZFP30 |  |  | 3732 | -0.451 | 0.1377 | No |
| 244 | PCLO |  |  | 3753 | -0.456 | 0.1360 | No |
| 245 | BZW2 |  |  | 3865 | -0.480 | 0.1163 | No |
| 246 | SPATS2 |  |  | 4033 | -0.521 | 0.0856 | No |
| 247 | EPS8 |  |  | 4072 | -0.531 | 0.0807 | No |
| 248 | MAPRE2 |  |  | 4160 | -0.557 | 0.0661 | No |
| 249 | SNRNP40 |  |  | 4180 | -0.562 | 0.0652 | No |
| 250 | CACNA2D1 |  |  | 4302 | -0.598 | 0.0440 | No |
| 251 | FAM13A |  |  | 4397 | -0.634 | 0.0285 | No |
| 252 | FMNL2 |  |  | 4489 | -0.673 | 0.0137 | No |
| 253 | SYT1 |  |  | 4552 | -0.707 | 0.0049 | No |
| 254 | HS6ST1 |  |  | 4554 | -0.707 | 0.0082 | No |
| 255 | EIF2B3 |  |  | 4602 | -0.734 | 0.0025 | No |
| 256 | EFNB2 |  |  | 4610 | -0.738 | 0.0049 | No |
| 257 | MIAT |  |  | 4622 | -0.748 | 0.0064 | No |
| 258 | MCTP1 |  |  | 4624 | -0.748 | 0.0100 | No |
| 259 | NT5C3A |  |  | 4664 | -0.771 | 0.0061 | No |
| 260 | EZR |  |  | 4714 | -0.803 | 0.0003 | No |
| 261 | SEMA3C |  |  | 4725 | -0.811 | 0.0024 | No |
| 262 | PAK1 |  |  | 4776 | -0.842 | -0.0033 | No |
| 263 | SNHG8 |  |  | 4826 | -0.893 | -0.0086 | No |
| 264 | L1CAM |  |  | 4924 | -0.992 | -0.0230 | No |
| 265 | SMURF2 |  |  | 4925 | -0.992 | -0.0180 | No |
| 266 | SLC38A1 |  |  | 4971 | -1.043 | -0.0217 | No |
| 267 | DDX10 |  |  | 5008 | -1.109 | -0.0233 | No |
| 268 | CLMP |  |  | 5009 | -1.111 | -0.0178 | No |
| 269 | LBH |  |  | 5038 | -1.168 | -0.0175 | No |
| 270 | NTM |  |  | 5052 | -1.188 | -0.0141 | No |
| 271 | SMS |  |  | 5109 | -1.315 | -0.0187 | No |
| 272 | SORBS2 |  |  | 5124 | -1.342 | -0.0147 | No |
| 273 | CHD3 |  |  | 5131 | -1.364 | -0.0090 | No |
| 274 | NRP1 |  |  | 5177 | -1.553 | -0.0102 | No |
| 275 | TLE3 |  |  | 5206 | -1.684 | -0.0073 | No |
| 276 | THBS1 |  |  | 5222 | -1.800 | -0.0013 | No |
| 277 | EDIL3 |  |  | 5282 | -2.918 | 0.0016 | No |
Table: GSEA details [plain text format]

  

Fig 2: RICHARDS\_NOWAKOWSKI\_SCIENCE\_2017\_NEN\_EARLY2\_UPREG: Random ES distribution      
 Gene set null distribution of ES for **RICHARDS\_NOWAKOWSKI\_SCIENCE\_2017\_NEN\_EARLY2\_UPREG**
